# PReS-FINAL-2060: The role of stress factors in the onset of juvenile idiopathic arthritis

**DOI:** 10.1186/1546-0096-11-S2-P72

**Published:** 2013-12-05

**Authors:** N Stepanenko, T Shelepina, I Nikishina

**Affiliations:** 1 V.A. Nasonova Research Institute of Rheumatology, Moscow, Russian Federation

## Introduction

According to the data found in the literature on clinical psychology, RA refers to the group of psychosomatic diseases, the leading role in occurrence of which is played by negative life events that cause stress.

## Objectives

To compare the frequency of negative life events in the areas of family, education, communication with peers in patients with LA before the occurrence of the disease and in a control group of nominally healthy children.

## Methods

The biographical methodology of "The life line", the picture of the family, drawings "I am at school", "Me and my friend" and a clinical interview.

## Results

**Table 1:** 

Areas	The main group comprised 30 patients with various LA at the age of 7 to 13 years (in all, 152 events)				The control group of nominally healthy children with no articular pathology comprised 10 persons aged between 8 and 14 years old (in all, 76 events)			
	**Positive events**		**Negative events**		**Positive events**		**Negative events**	

Family	48	31.6%	94	**61.8** **% ***P *= 0.000	23	30%	16	21%

Education	34	22.4%	10	6.6%	12	15.8%	7	9.2%

Communication	9	5.9%	3	2%	10	13%	8	10%

## Conclusion

A statistically significant increase in the frequency of family stress in patients in the period preceding the onset of LA was revealed.

## Disclosure of interest

None declared.

